# Decoding Severity in Crotalic Snakebite Cases: Findings From a Decade of Cohort Analysis in Brazil

**DOI:** 10.1155/bmri/7761982

**Published:** 2026-06-25

**Authors:** Luciana Reis da Silveira, Aloisio Joaquim Freitas Ribeiro, Victor Schulthais Chagas, Patryk Marques da Silva Rosa, Julia Alpino de Castro Silva, Victoria Fialho Baroni Neves, Adebal de Andrade Filho, Milena Soriano Marcolino

**Affiliations:** ^1^ Programa de Pós-Graduação em Ciências da Saúde: Infectologia e Medicina Tropical, Faculty of Medicine, Universidade Federal de Minas Gerais, Belo Horizonte, Minas Gerais, Brazil, ufmg.br; ^2^ João XXIII Hospital, Emergency Hospital Complex of the Hospital Foundation of the State of Minas Gerais (FHEMIG), Belo Horizonte, Minas Gerais, Brazil; ^3^ Faculdade de Ciências Médicas de Minas Gerais, Lucas Machado Educational Foundation (FELUMA), Belo Horizonte, Minas Gerais, Brazil; ^4^ Department of Statistics, Universidade Federal de Minas Gerais, Belo Horizonte, Minas Gerais, Brazil, ufmg.br; ^5^ Telehealth Center, University Hospital, Universidade Federal de Minas Gerais, Belo Horizonte, Minas Gerais, Brazil, ufmg.br; ^6^ Medical School, Universidade Federal de Viçosa, Viçosa, Minas Gerais, Brazil, ufv.br; ^7^ University Hospital, Universidade Federal de Minas Gerais, Belo Horizonte, Minas Gerais, Brazil, ufmg.br; ^8^ Institute for Health Assessment and Translation for Chronic and Neglected Diseases of High Relevance (IATS-CARE), Belo Horizonte, Brazil

**Keywords:** *Crotalus*, epidemiology, signs and symptoms, snakebites

## Abstract

**Background:**

Crotalic snakebite accidents are a common cause of admission into toxicology units in Brazil, and are associated with substantial morbidity and mortality. Our aim was to analyze clinical and laboratory findings, outcomes, and variables associated with the severity of patients treated for crotalic accidents at a reference center in Brazil.

**Methods:**

This retrospective cohort study included consecutive patients treated at a referral center in Minas Gerais state, Brazil, between January 2011 and December 2022. Data were collected from medical records. Descriptive analyses, as well as univariate and multivariate logistic regression analyses, were performed.

**Results:**

A total of 415 patients were included (median age 41; interquartile range [IQR] 23–56; 79.5% male). Of these, 67.5% were admitted within 6 h of the incident, and 73.7% of the incidents occurred in rural areas. The median length of hospital stay was 3 days (IQR 2–4); 44.1% of the cases were identified as severe, and four patients died. Factors associated with severity included age ≤ 20 years (OR 2.99; 95% CI 1.65–5.50) or ≥ 50 years (OR 1.79; 95% CI 1.08–2.96), unidentified or unconfirmed snake species (OR 4.18; 95% CI 2.06–8.77 and OR 2.60; 95% CI 1.54–4.44, respectively), and treatment delays > 6 h (OR 4.04; 95% CI 2.46–6.72).

**Conclusion:**

This study highlights the burden of crotalic snakebite accidents and the need for targeted prevention and timely access to healthcare, particularly for rural populations. Addressing delays in treatment and improving snake identification can reduce the risk of severe outcomes.

## 1. Introduction

Snakebite envenomation is a neglected tropical disease and a significant public health problem in tropical and subtropical regions, with an estimated 5.4 million snakebites and 2.7 million envenomations reported annually worldwide. These incidents disproportionately affect rural populations in Africa, Asia, Oceania, and Latin America, leading to physical and psychological sequelae, amputations, permanent disabilities, and fatalities [1–4]. In Brazil, where snakebites have been a mandatory notifiable condition since 2010, over 32,000 incidents were reported in 2023 [5], with a fatality rate of 0.32% described in the year before that [6], reflecting the persistent burden and the need for effective preventive and management strategies.

Among venomous snakebites in Brazil, those caused by *Crotalus durissus* species (rattlesnakes) represent approximately 7.7% of all reported incidents, with a geographic distribution reaching 30% in certain regions. These bites, referred to as crotalic accidents, are characterized by mild local symptoms and potentially severe systemic manifestations, including neuromuscular paralysis, rhabdomyolysis, coagulopathy, and renal dysfunction [7–12]. Fatal outcomes may occur in severe cases [9, 8]. The venom′s neurotoxic, myotoxic, coagulant, and nephrotoxic properties underline the need for timely administration of crotalic antivenom, the only effective treatment available [8]. This approach is of great relevance for public health, given the global notoriety of this condition, which significantly impacts different populations [1, 13].

Brazil harbors a single rattlesnake species, *C. durissus*, with several subspecies distributed across diverse regions. In the state of Minas Gerais, the predominant subspecies are *C. durissus terrificus* and *C. durissus collilineatus*. Differences in venom composition, snake behavior, and ecology across subspecies may influence the clinical presentations and severity of accidents. These variations highlight the importance of epidemiological studies to guide prevention strategies, resource allocation, and treatment protocols in high‐risk areas. [7, 14–17]

The Toxicological Information and Assistance Center of Minas Gerais (CIATox‐MG) is one of Brazil′s largest referral centers for toxicology. It is a reference for the whole state of Minas Gerais, Southeast Brazil, the fourth largest Brazilian state by area and the second largest in number of inhabitants. Between 2019 and 2023, CIATox‐MG recorded over 121,000 consultations, of which 27,774 were in‐person, demonstrating its critical role in managing envenomation cases [18, 19]. Therefore, this study is aimed at characterizing the epidemiological, clinical, and laboratory profiles of patients treated for crotalic accidents at CIATox‐MG. Additionally, it seeks to evaluate outcomes such as severity, length of hospital stay, and complications, while exploring factors associated with case severity.

## 2. Methods

### 2.1. Study Design and Participants

This cohort study adheres to the Strengthening the Reporting of Observational Studies in Epidemiology (STROBE) guidelines (Supporting File S1) [20]. Consecutive patients of any age range and sex, treated in person at CIATox‐MG, from January 1, 2011, to December 31, 2022, and classified as victims of crotalic envenomation were included. CIATox‐MG is located at Hospital João XXIII, in the city of Belo Horizonte, the capital of Minas Gerais state, Brazil.

The list of patients was obtained from the registry in the compulsory notification system for venomous animal accidents–SINAN (*Sistema de Informações de Agravos de Notificação–Notifiable Diseases Information System*), and from the list obtained from the Brazilian Poisoning Data System of the Toxicological Information and Assistance Centers (DATATOX) [21].

Crotalic envenomation was defined as any case of suspected or confirmed contact with a rattlesnake, evidenced by clinical, epidemiological, or laboratory findings. Epidemiological suspicion was determined based on the reported location of contact, particularly rocky areas and regions with a history of rattlesnake envenomation. Epidemiological confirmation was based on medical records documenting direct observation of the animal (live or dead), a photograph, or the description of rattlesnake‐specific characteristics, combined with local or systemic clinical manifestations and supportive laboratory findings, following SINAN notification criteria [22]. While identifying the snake is ideal for classifying a crotalic envenomation, in clinical practice, many patients are unable to capture the snake responsible, either because the animal escapes or because the circumstances of the bite do not allow for proper identification. In these cases, the diagnosis relies on standardized clinical, laboratory (Table 1), and epidemiological criteria, which are well established in the literature and widely applied by healthcare professionals [23]. These criteria ensure a reliable classification of crotalic envenomation, even in the absence of direct identification of the snake, as documented in the medical records. In the study region, *C. durissus* may share its ecological niche with other medically important venomous snakes, particularly species of the genus *Bothrops* and, less frequently, *Micrurus* spp. However, crotalic envenomation typically presents a characteristic clinical syndrome including neurotoxicity, systemic myolysis, and myoglobinuria, which differs from the clinical manifestations of these other envenomations. Therefore, in the absence of laboratory confirmation, cases were classified based on clinical syndromic criteria.

**Table 1:** Severity classification of crotalic envenomation.

Classification	Clinical and laboratory findings
Mild	Absence of eyelid ptosis and visual blurring or discrete late onset, without change in urine color, absent, or discrete myalgia, generally normal laboratory tests.
Moderate	Early onset mild eyelid ptosis and blurred vision, mild myalgia, dark urine, rhabdomyolysis, and mild coagulation test abnormalities (close to reference values, with no record of full anticoagulation)
Severe	Eyelid ptosis and visual blurring are evident and intense, intense and generalized myalgia, dark urine, oliguria or anuria, rhabdomyolysis is usually present, and coagulation tests are altered.

*Note:* This table was adapted from the investigation form for accidents involving venomous animals—Sinan Net [21] and Andrade Filho et al. [24].

Patients initially listed as suspected crotalic envenomation, but without clinical or laboratory confirmation of the diagnosis during hospital care, or follow‐up visits unrelated to the initial hospitalization were excluded.

### 2.2. Variables and Data Sources

The following data were obtained:

• **Sociodemographic data**: sex (male or female), age (years), and race;
• **Envenomation characteristics**: location (city), month/year, time between the snakebite and care (categorized as 0–1, 1–3, 3–6, 6–12, 12–24, over 24 h, and unknown), location of the bite (head or trunk, upper limbs, and lower limbs), whether the animal was identified, and whether the envenomation was work related;
• **Clinical data**: occurrence and types of local and systemic manifestations, crotalic antivenom therapy;
• **Local manifestations:** pain, edema, bruising, necrosis, and others;
• **Systemic manifestations:** neuroparalytic (e.g., eyelid ptosis and blurred vision), hemorrhagic (e.g., gingival bleeding and other bleeding), vagal (e.g., vomiting and diarrhea), myolytic/hemolytic (e.g., myalgia, anemia, and dark urine), renal, and others;
• **Laboratory data**: values on admission and maximum of total creatine phosphokinase (CK), serum creatinine test, international normalized ratio (INR), prothrombin time, values on admission and minimum of prothrombin activity, hemoglobin, platelets, and fibrinogen;
• **Outcomes**: classification of envenomation severity as mild, moderate, and severe (Table 1), length of hospital stay, local complications, systemic complications, and death.

Distances between the city of occurrence and the address of CIATox‐MG, at Hospital João XXIII were calculated using Google Maps [23, 25]. To ensure reproducibility, estimates were based on road distance (driving mode) rather than straight‐line (Euclidean) distance. In the absence of information on the exact route taken by each patient, the shortest road route was used as the standard parameter for the epidemiological analysis of geographic accessibility and health‐seeking delay. Renal manifestations included any clinical signs or symptoms related to kidney involvement, such as oliguria, anuria, and other changes in urinary appearance, as well as renal failure. Renal failure was specifically defined as a decline in kidney function or the need for dialysis, as documented in the medical records.

Envenomation severity was classified as outlined in Table 1, based on medical records or assessments performed by the researcher (L.R.S.). The definitions of local and systemic complications are provided in Table S1.

The antivenom provided at Hospital João XXIII is produced by the major Brazilian serum laboratories following national protocols. The ampoules of crotalic serum available at the institution during the study period were produced by three major official Brazilian laboratories: Butantan, Vital Brazil, and Fundação Ezequiel Dias (Funed). Since 2017, Butantan′s antivenom has been the most available and officially registered at the Toxicology Unit [26]. The Ministry of Health is the responsible agency for acquiring and distributing the serum to state health departments, which then forward it to reference hospital centers for protocol use in cases of envenomation involving animals [27].

The crotalic antivenom is a heterologous immunoglobulin against venom from *Crotalus* genus snakes. It is presented in 10‐mL vials of intravenous injectable solution containing the F(ab ^′^)2 fraction of purified specific immunoglobulins, obtained from the plasma of equines hyperimmunized with a mixture of crotamine‐positive venoms, along with excipients such as phenol, sodium chloride, and water for injectable solutions. Serum neutralization tests in mice estimate that each milliliter of serum neutralizes at least 1.5 mg of *C. durissus* ssp venom, totaling at least 15.0 mg per vial. Its effect begins immediately after infusion, neutralizing toxins from *Crotalus* genus snake venoms that are circulating and, possibly, in the tissues. The antigen–antibody reaction occurs during the specific treatment in which the F(ab ^′^)2 fraction of specific immunoglobulins binds to the venom toxins, neutralizing them [27–29].

There is a consensus that early specific care is directly related to greater therapeutic potential. Mild cases receive five ampoules of crotalic serum, moderate cases receive 10 ampoules, and severe cases receive 20 ampoules. The classification is dynamic during hospitalization. If clinical or laboratory conditions worsen, the specific antivenom serum therapy should be supplemented, along with all appropriate supportive treatments to reduce damage [7].

Data were collected through CIATox‐MG′s Hospital Information and Management System (SIGH), using codes X20 and T630 from the International Classification of Diseases (ICD‐10) [30] and verified with SINAN records. For patients without digital medical records (due to the adaptation to the hospital′s transition to the digital medical record model in 2011 and possible system instabilities), data from SINAN forms were used. Data collection was performed by a team of trained researchers (physician and medical students) and validated for accuracy.

### 2.3. Statistical Analysis

Descriptive analyses were conducted for all study variables. Categorical variables were summarized as counts and percentages, whereas continuous variables were presented as means with standard deviation or medians with interquartile ranges, depending on the data distribution. Laboratory test results were categorized based on the hospital′s reference values, certified by ISO 9001/2015: INR (0.91–1.25), prothrombin (normal > 70%), hemoglobin (normal 13.5–17.5 mg/dL), platelets (normal 150,000–450,000/mm^3^) and fibrinogen (normal 200–393 mg/dL).

**Figure 1:**
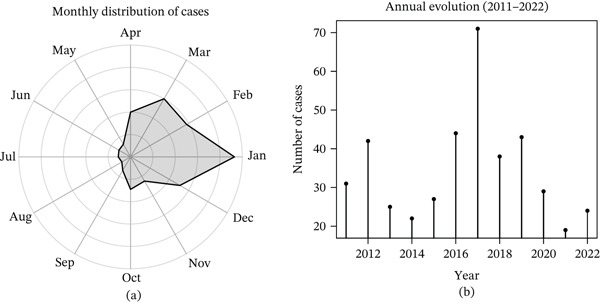
(a) Distribution of cases by month of occurrence; (b) evolution of the number of crotalic envenomation patients attended from 2011 to 2022.

**Figure 2:**
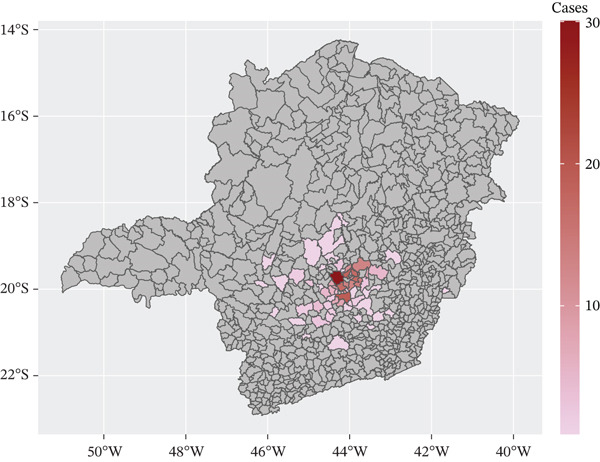
Distribution map by location of occurrence of crotalic envenomation treated at CIATox‐MG at Hospital João XXIII between 2011 and 2022.

Temporal and geographic distributions of envenomation were described using a radar chart and frequency map, respectively (Figures 1 and 2).

Logistic regression models were used to identify factors associated with severe crotalic envenomation. Potentially predictive variables included: age, sex, race/color, identification of the animal, time upon hospital admission, and area where the envenomation occurred.

Age was categorized into three groups (< 20, 20–49, and ≥ 50 years) to reflect different exposure patterns and vulnerability profiles. Time to treatment was dichotomized at 6 h (< 6 vs. ≥ 6 h), as delayed antivenom administration beyond this period has been associated with an increased risk of systemic complications in snakebite envenomation.

Laboratory data were not included in the logistic regression analysis, as there is a time interval between the snakebite and patient admission. Given this delay, laboratory parameters may reflect the progression of envenomation rather than serve as true predictors of severity at hospital presentation.

Missing data were handled using the missing indicator method, in which missing values were treated as a separate category in the regression models. Treating missing values as a separate category allowed us to retain these observations in the model while accounting for potential systematic differences. In addition, this approach allowed the inclusion of all cases in the analysis and avoided the reduction in sample size associated with complete‐case analysis.

The complete model follows the theoretical framework of logistic regression, widely established in the literature. The logistic regression model for *Y*_*i*_, the indicator variable for severe accidents (I(accident severity=severe), was defined as:

logitPYi=1Xi=β0+β1X1i+⋯+βpX1p,

where *X*_*i*_ = *c*(*X*_1*i*_, ⋯., *X*_*p* *i*_) is the vector of predictor variables for the *i*‐th observation, consisting of indicator (dummy) variables representing the categories of each categorical predictor; *β*_0_ is the model intercept, and *β* = (*β*_1_, ⋯, *β*_*p*_) is the vector of regression coefficients corresponding to the predictors in *X*_*i*_.

Univariable logistic regression models were first fitted for each predictor using the first category as the reference. The “NI” (not informed) category was treated as a separate level for each categorical variable to preserve sample size and assess its potential effect on the outcome. Predictors with *p* values ≤ 0.20 in univariable analysis were included in the multivariable logistic regression model, following the criterion proposed by Bursac et al. [31]. Associations were quantified using odds ratios (ORs) with 95% confidence intervals (CIs).

### 2.4. Ethical Aspects

This study was approved by the Research Ethics Committee of the Hospital Foundation of the State of Minas Gerais (CEP‐FHEMIG), to which Hospital João XXIII belongs, under opinion: 5,261,707 (Certificate of Presentation of Ethical Review–CAAE 03815912.4.0000.5119). Given that data were obtained exclusively from medical records and patient de‐identification was ensured, the requirement for informed consent was waived.

## 3. Results

During the study period, 433 consecutive patients who were victims of crotalic envenomation were seen at the Toxicology Unit of Hospital João XXIII. Of these, 415 were included in the present study. Three patients were excluded due to secondary infection treatment and 15 cases due to lack of diagnostic confirmation (Figure S1).

### 3.1. Envenomation Cases: Time and Location

Seasonal trends were observed in the frequency of crotalic envenomation, with fewer cases recorded from May to September and peak numbers occurring between 2016 and 2019 (Figure 1a,b).

Most cases occurred within a 100‐km radius of Belo Horizonte, the capital city of Minas Gerais state, with only four cases from more distant municipalities (Figure 2). Esmeraldas (7.2%) and Ribeirão das Neves (6.0%) were the cities with the highest incidence of cases, 56.1 and 32.29 km away from CIATox‐MG, respectively. Rural areas accounted for 73.7% of the cases. The distribution of cases between rural and urban areas stratified by year is shown in Figure S3.

### 3.2. Demographic Characteristics of Patients

The mean age of patients was 40 ± 20 years, ranging from 1 to 86 years. Notably, 10.8% of cases involved children under 12 years, and 18.8% involved elderly individuals (≥ 60 years). The majority of the patients were male (79.5%) and *pardos* (69.6%). Detailed demographic data are summarized in Table 2.

**Table 2:** Characteristics of patients and crotalic envenomation, 2011–2022 (*n* = 415).

Variable	*N* (%)
Age range	
0–9 years	35 (8.4)
10–19 years	50 (12.1)
20–29 years	49 (11.8)
30–39 years	66 (15.9)
40–49 years	66 (15.9)
50–59 years	71 (17.1)
60–69 years	52 (12.5)
70–79 years	20 (4.8)
80–89 years	6 (1.5)
Men	330 (79.5)
Race	
*Pardos*	289 (69.6)
Caucasian	91 (21.9)
Black	21 (5.1)
Asian	1 (0.2)
Indigenous	0 (0)
Not informed	13 (3.1)
Envenomation related to work	
No	299 (72.0)
Yes	90 (21.7)
Not informed	26 (6.3)
Time upon hospital admission	
0–1 h	37 (8.9)
1–3 h	141 (34.0)
3–6 h	102 (24.6)
6–12 h	54 (13.0)
12–24 h	34 (8.2)
> 24 h	32 (7.7)
Not informed	15 (3.6)
Location of the bite	
Lower limbs	285 (68.7)
Upper limbs	78 (18.8)
Trunk or head	51 (12.3)
Not informed	1 (0.2)
Identification of the snake	
Yes, with confirmation	168 (40.5)
Yes, but without confirmation	122 (29.4)
Nonidentified	59 (14.2)
Not informed	66 (15.9)
Zone of occurrence of the envenomation	
Rural areas	306 (73.7)
Urban areas	75 (18.1)
Periurban areas^a^	19 (4.6)
Not informed	15 (3.6)

^a^Peri‐urban areas: areas on the outskirts of urban centers.

### 3.3. Envenomation Characteristics and Clinical Manifestations

The majority of the crotalic bites occurred in the lower limbs (68.7%), followed by the upper limbs (18.8%). Work‐related activities were associated with 21.7% of cases. Only 8.9% of patients received treatment within 1 h of the bite, whereas 58.6% sought care between 1 and 6 h (Table 2).

Local manifestations upon admission were reported in 374 (90.1%) of the cases, with pain being the most common manifestation (80.7%), followed by edema (55.6%). In 308 (74.2%) cases, there was at least one systemic manifestation. Neuroparalytic manifestations were the most common (67.2%), followed by myolytic or hemolytic (37.1%), vagal (19.8%), renal (17.1%), and hemorrhagic manifestations (5.8%) (Table 3).

**Table 3:** Clinical and laboratorial findings 2011–2022 (*n* = 415).

Variable	*N* (%)
Laboratorial findings at admission	
Fibrinogen < 200 mg/dL	284 (68.4)
INR > 1.25	202 (48.7)
Prothrombin activity < 70%	198 (47.7)
Hemoglobin < 13.5 mg/dL	133 (32.1)
Platelets < 150,000/mm^3^	46 (11.1)
Presence of local manifestations	
Yes	374 (90.1)
No	38 (9.2)
Not informed	3 (0.7)
Local manifestations (*n* = 374)^a^	
Pain	335 (80.7)
Edema	231 (55.7)
Bruising	27 (6.5)
Necrosis	2 (0.5)
Other types of local manifestations^b^	180 (43.4)
• Inflammatory signs	108 (28.9)
• Neurosensory	104 (27.8)
• Local manipulation/(tourniquet)	25 (6.7)/15 (4.0)
• Bleeding	11 (2.9)
Presence of systemic manifestations	
Yes	308 (74.2)
No	104 (25.1)
Not informed	3 (0.7)
Systemic manifestation (*n* = 308)^c^	
Neuroparalitics	279 (67.2)
Miolicits/hemolitics	154 (37.1)
Renal	71 (17.1)
Vagal	82 (19.8)
Hemorrhagic	24 (5.8)
Other systemic manifestations	121 (29.2)

^a^There are cases in which more than one alteration is present, the sum of the % exceeds 100%.

^b^Other local manifestations, under the described groupings, may exceed 100% since a patient may present several of the following: blisters, heat, ocher dermatitis, erythema, rash, phlycten, hematoma, hyperemia, ipsilateral livedo reticularis, and flushing, in addition to neuropathic manifestations: anesthesia, numbness, tingling, hypoesthesia, hyperesthesia, burning, paresthesia and manipulation of the site with scratches, abrasions, cuts, and bleeding, suction of the lesion and tourniquet.

^c^Other systemic manifestations, under groupings, may exceed 100% since a patient may present several alterations.

### 3.4. Laboratory Findings

Patients′ laboratory profile is shown in Table S2, laboratory abnormalities are shown in Table 3, and their stratification according to case severity is shown in Table 4. Common findings at hospital admission included fibrinogen levels < 200 mg/dL (68.4%), INR > 1.25 (48.7%), and prothrombin activity < 70% (47.7%). The proportion of abnormal laboratory parameters increased as case severity progressed, from mild to moderate and severe cases.

**Table 4:** Frequency distribution of case severity for the different categories of the variables of interest.

Variable	Categories	Case severity		
		Mild *n* (%)	Moderate *n* (%)	Severe *n* (%)
*Demographic*				
Sex	Women	20 (23.5)	32 (37.6)	33 (38.8)
	Men	73 (22.1)	107 (32.4)	150 (45.5)

Age (years)	[0, 10)	4 (11.4)	8 (22.9)	23 (65.7)
	[10, 20)	10 (20.0)	15 (30.0)	25 (50.0)
	[20, 30)	15 (30.6)	17 (34.7)	17 (34.7)
	[30, 40)	17 (25.8)	22 (33.3)	27 (40.9)
	[40, 50)	20 (30.3)	28 (42.4)	18 (27.3)
	[50, 60)	16 (22.5)	20 (28.2)	35 (49.3)
	[60, 70)	10 (19.2)	21 (40.4)	21 (40.4)
	[70, 80)	1 (5.0)	7 (35.0)	12 (60.0)
	[80, 90)	0 (0.0)	1 (16.7)	5 (83.3)

*Envenomation characteristics*				
Location of the bite^a^	Lower limbs	13 (25.5)	18 (35.3)	20 (39.2)
	Upper limbs	19 (24.4)	28 (35.9)	31 (39.7)
	Trunk or head	61 (21.3)	93 (32.5)	132 (46.2)

Time until hospital admission (hours)	0–1	9 (24.3)	14 (37.8)	14 (37.8)
	1–3	37 (26.2)	59 (41.8)	45 (31.9)
	3–6	30 (29.4)	36 (35.3)	36 (35.3)
	6–12	7 (13.0)	10 (18.5)	37 (68.5)
	12–24	3 (8.8)	5 (14.7)	26 (76.5)
	≥ 24	3 (9.4)	7 (21.9)	22 (68.8)
	Ignored	4 (26.7)	8 (53.3)	3 (20.0)

Identification of the snake	Identified	58 (34.5)	57 (33.9)	53 (31.5)
	Identified, but not confirmed	18 (14.8)	39 (32.0)	65 (53.3)
	Nonidentified	3 (5.1)	15 (25.4)	41 (69.5)
	Not informed	14 (21.2)	28 (42.4)	24 (36.4)

Zone of occurrence	Urban	20 (26.7)	26 (34.7)	29 (38.7)
	Rural	65 (21.2)	95 (31.0)	146 (47.7)
	Peri‐urban^b^	3 (15.8)	13 (68.4)	3 (15.8)
	Not informed	5 (33.3)	5 (33.3)	5 (33.3)
Envenomation related to work	Yes	26 (28.9)	28 (31.1)	36 (40.0)
	No	63 (21.1)	104 (34.8)	132 (44.1)
	Not informed	4 (15.4)	7 (26.9)	15 (57.7)

*Laboratory*				
CK at admission according to quartiles (U/L)	≤ 173	45 (46.4)	35 (36.1)	17 (17.5)
	[173, 399)	25 (26.6)	47 (50.0)	22 (23.4)
	[399, 3475)	16 (16.8)	35 (36.8)	44 (46.3)
	≥ 3475	1 (1.0)	10 (10.4)	85 (88.5)
	Not informed	6 (18.2)	12 (36.4)	15 (45.5)

Creatinine at admission according to quartiles (mg/dL)	≤ 0.66	12 (13.2)	27 (29.7)	52 (57.1)
	[0.66, 0.81)	24 (26.4)	30 (33.0)	37 (40.7)
	[0.81, 0.94)	24 (26.1)	35 (38.0)	33 (35.9)
	≥ 0.94	16 (16.7)	33 (34.4)	47 (49.0)
	Not informed	17 (37.8)	14 (31.1)	14 (31.1)

INR	≤ 1.25	65 (36.3)	74 (41.3)	40 (22.3)
	> 1.25	23 (11.4)	52 (25.7)	127 (62.9)
	Not informed	5 (14.7)	13 (38.2)	16 (47.1)

Prothrombin activity (%)	≤ 70	66 (36.1)	76 (41.5)	41 (22.4)
	> 70	22 (11.1)	50 (25.3)	126 (63.6)
	Not informed	5 (14.7)	13 (38.2)	16 (47.1)

Hemoglobin (g/dL)	13.5–17.5	57 (24.2)	67 (28.4)	112 (47.5)
	< 13.5	27 (20.3)	52 (39.1)	54 (40.6)
	Not informed	4 (15.4)	7 (26.9)	15 (57.7)

Platelets (cells/mm^3^)	150,000–450,000	78 (24.1)	108 (33.4)	137 (42.4)
	< 150,000	6 (13.0)	11 (23.9)	29 (63.0)
	Not informed	9 (19.6)	20 (43.5)	17 (37.0)

Fibrinogen (mg/dL)	200–393	39 (44.8)	29 (33.3)	19 (21.8)
	< 200	48 (16.6)	95 (32.8)	147 (50.7)
	Not informed	6 (15.8)	15 (39.5)	17 (44.7)

*Note:* Reference values: INR: 0.91–1.25; fibrinogen: 200–393 mg/dL; hemoglobin: 13.5–17.5 g/dL; platelets: 150,000–450,000 cells/mm^3^; prothrombin activity: above 70%.

Abbreviations: CK, creatine phosphokinase; INR, international normalized ratio.

^a^For an observation with an unknown location, the lower limb category was imputed as the mode of the distribution.

^b^Peri‐urban areas: areas on the outskirts of urban centers.

### 3.5. Case Evolution and Outcomes

The median hospital stay was 3 days (IQR 2–4 days; Figure S2). Local complications occurred in 3.4% of cases. Systemic complications, such as renal failure (7.7%) and respiratory failure (3.9%), were observed in 8.4% of patients (Table 5).

**Table 5:** Frequency of outcomes of the crotalic envenomation 2011–2022 (*n* = 415).

Variable	*N* (%)
Case severity	
Mild	93 (22.4)
Moderate	139 (33.5)
Severe	183 (44.1)
Laboratory outcomes	
Fibrinogen < 200 mg/dL	308 (74.2)
INR > 1.25	249 (60.0)
Prothrombin activity < 70	242 (57.8)
Hemoglobin < 13.5 mg/dL	200 (48.2)
Platelets < 150,000 cells/mm^3^	70 (16.9)
Local complications	(*n* = 14)^a^
Infection	14 (3.4)
Function deficit	4 (1.0)
Enucleation	1 (0.2)
Systemic complications	(*n* = 35)^a^
Renal failure	32 (7.7)
Respiratory failure	16 (3.9)
Shock	9 (2.2)
Sepsis	4 (1.0)
Use of crotalic antivenom	
Yes	382 (92.1)
No	30 (7.3)
Not informed	3 (0.7)
Final outcome	
Discharged alive	407 (98.1)
Death by venomous animal	4 (1.0)
Death by other causes	1 (0.2)
Not informed	3 (0.7)

^a^For systemic and local complications, there are seven missing cases.

Cure was achieved in 98.1% of cases, with five fatalities recorded (1.2%). Four patients who died were men, two were *pardos*, median age 79.5 years, all came from cities less than 270 km from Belo Horizonte, two referred crotalic envenomation cases in rural areas, three were bitten on the feet, and all four patients took more than 6 h to receive specific treatment. All of them were reported before 2017 had local and systemic manifestations of crotalic envenomation. Three had acute renal failure reported, two severe rhabdomyolysis (admission and maximum of total CK above 140.000 U/L). It was not possible to determine the immediate cause of these deaths in the hospital. According to Brazilian regulations, all patients who die from external causes must be evaluated by an expert from the forensic medical institute; the death certificate report is confidential and does not form part of the patient′s hospital medical record.

### 3.6. Variables Associated With Severe Crotalic Envenomation

Table 4 presents the frequency distributions of case severity (mild, moderate, or severe) according to the following categories of variables: age, sex, snake identification, bite location, area of occurrence, and time between the snakebite and hospital care. Table 5 shows the frequency of outcomes of the crotalic envenomation.

The full model specification for severe crotalic envenomation, including variable coding, reference categories, and the results of univariate and multivariate logistic regression analyses is detailed in Table 6. Table S3 presents the results of the multiple logistic regression model for case severity. The severity of crotalic envenomation (mild, moderate, or severe) was associated with: (i) time to treatment: admission > 6 h postenvenomation increased severity risk (OR 4.04; 95% CI 2.46–6.72); (ii) age: higher severity in patients aged 0–20 years (OR 2.99; 95% CI 1.65–5.50) and ≥ 50 years (OR 1.79; 95% CI 1.08–2.96); and (iii) snake identification: unidentified or nonconfirmed identification raised severity risk (OR 4.18; 95% CI 2.06–8.77).

**Table 6:** Univariate and multivariate analysis of the variables associated with the occurrence of serious crotalic envenomation.

Variable	Categories	Severe cases		Univariate logistic regression		Multivariate logistic regression	
		No *n* (%)	Yes *n* (%)	*p* value ^∗^	Odds ratio (95% CI)	*p* value ^∗^	Odds ratio (CI 95)
Sex	Women	52 (61.2)	33 (38.8)	0.2730	1	0.9600	1
	Men	73 (22.1)	107 (32.4)		1.31 (0.81; 2.15)		1.01 (0.58; 1.79)

Age	[20, 50)	37 (43.5)	48 (56.5)	0.0009	1	0.0008	1
	[0,20)	119 (65.7)	62 (34.3)		**2.49 (1.47; 4.24)**		**2.99 (1.65; 5.50)**
	[50,90]	76 (51.0)	73 (49.0)		**1.84 (1.18; 2.88)**		**1.79 (1.08; 2.96)**

Location of the bite^a^	Trunk or head	31 (60.8)	20 (39.2)	0.4512	1	NA	NA
	Upper limbs	47 (60.3)	31 (39.7)		1.02 (0.50; 2.12)		
	Lower limbs	153 (53.8)	132 (46.2)		1.33 (0.73; 2.47)		

Time until hospital admission	< 6 h	185 (66.1)	95 (33.9)	< 0.0001	1	< 0.0001	1
	≥ 6 h	35 (29.2)	85 (70.8)		**4.73 (2.99; 7.60)**		**4.04 (2.46; 6.72)**
	Not informed	12 (80.0)	3 (20.0)		0.49 (0.11; 1.58)		0.46 (0.10; 1.64)

Identification of the snake	Identified	115 (68.5)	53 (31.5)	< 0.0001	1	< 0.0001	1
	Identified, but not confirmed	57 (46.7)	65 (53.3)		**2.47 (1.53; 4.02)**		**2.60 (1.54; 4.44)**
	Non identified	18 (30.5)	41 (69.5)		**4.94 (2.64; 9.58)**		**4.18 (2.06; 8.77)**
	Not informed	42 (63.6)	24 (36.4)		1.24 (0.68; 2.24)		1.51 (0.76; 2.98)

Zone of occurrence	Urban	46 (61.3)	29 (38.7)	0.0170	1	0.0682	1
	Peri‐urban^b^	16 (84.2)	3 (15.8)		**0.30 (0.06; 0.99)**		1.48 (0.83; 2.70)
	Rural	160 (52.3)	146 (47.7)		1.45 (0.87; 2.45)		0.36 (0.07; 1.33)
	Not informed	10 (66.7)	5 (33.3)		0.79 (0.23; 2.47)		0.80 (0.20; 2.84)

Envenomation related to work	Yes	54 (60.0)	36 (40.0)	0.2795	1	NA	NA
	No	167 (55.9)	132 (44.1)		1.19 (0.74; 1.92)		
	Not informed	11 (42.3)	15 (57.7)		2.05 (0.85; 5.06)		

*Note:* The category “Not Informed” represents data that were not provided in the original records and were included as a separate level to maintain model stability and sample size. The first category listed for each variable was considered the reference category. Categorical variables were incorporated into the model using dummy coding (indicator variables).

Abbreviations: CI, confidence interval; CK, creatine phosphokinase; NA, not applicable (not included in the model).

^a^For the only observation with unknown bite site, the lower limbs category was imputed as the mode of the distribution.

^b^Peri‐urban areas: areas on the outskirts of urban centers.

^∗^Likelihood ratio test.

## 4. Discussion

The results of this study provide a comprehensive overview of crotalic envenomation in a reference center in Minas Gerais, Brazil, emphasizing the importance of adequate reporting and treatment. Key findings include the predominance of crotalic envenomation among the economically active population (20–59 years), delayed hospital admissions (32.5% occurred over 6 h postbite), and a high proportion of cases in rural areas (73.7%). Severe cases were significant, with 44.1% identified as severe and four deaths reported. Factors associated with severity included age (≤ 20 years or ≥ 50 years), delays in treatment (> 6 h), and lack of snake identification, each significantly increasing the risk of complications.

Brazil has a high burden of snakebite envenomation, with an estimated incidence of 15.4–21.28 per 100,000 inhabitants. Globally, Brazil ranks third, tied with Vietnam [32]. A detailed understanding of the epidemiological, clinical, and laboratory aspects of crotalic envenomation is essential to assess the impact on public health and to understand the ecological aspects of these animals and to assess areas for improvement in patient care [8, 9, 33–35]. The present study aligns with previous research but adds specific data on crotalic envenomation.

Seasonality was evident, with 63.3% of cases occurring between December and March, consistent with increased snake activity during hot, rainy months, due to the abundance of food and the breeding season [36]. This period coincides with intensified agricultural activities, exposing rural workers to higher risks [37, 38]. This finding aligns with data from snakebite envenomation in general in the same region [39] or other regions of the country [40].

Although the economically active male population was most affected, extremes of age (≤ 20 and ≥ 50 years) showed greater severity, corroborating findings from studies on other snake genera [41]. For example, a study in the Amazon on envenomation caused by snakes of the genus *Micrurus* also observed greater severity at the extremes of age, with an OR of 1.26 (95% CI 1.03–1.52) for those under 15 years of age and OR of 1.53 (95% CI 1.09–2.13) for individuals aged 65 years or older [42], whereas in the present analysis, the OR was 2.99 (95% CI 1.65; 5.50) for those under 20 years of age and 1.79 (1.08; 2.96) for those 50 years or above. The age distribution of envenomation cases in our cohort showed a bimodal pattern, with higher frequencies among younger and older individuals. Similar patterns have been reported in other studies from Brazil [41, 42] and from Asian regions where snakebite is endemic, possibly reflecting different exposure dynamics, including recreational or peridomestic activities among younger individuals and increased vulnerability or rural exposure among older adults [43]. This emphasizes the critical importance of prioritizing timely care and preventive measures for individuals at the extremes of age. Further studies are needed to explore the underlying factors contributing to increased severity in these age groups, which may guide age‐specific prevention and management strategies.

Despite being a reference center, only about 9% of patients received medical care within 1 h of the incident, even though most of the cases treated were from locations less than 150 km away. This finding aligns with other studies in which there is a delay in seeking care [37, 44]. However, it contrasts with a study conducted in Mato Grosso state, located in Center‐West Brazil, in which 43.4% of cases were treated within 1 h after the snakebite [45]. There are challenges in accessing health care, specifically related to antivenom immunotherapy [46–49], highlighting the rational and timely use of resources, technical training [50], production [51], distribution, and treatment costs [52]. Delays significantly impacted severity, with a fourfold increase in severe cases when treatment was delayed beyond 6 h (OR 4.23; 95% CI 2.55–7.12). These findings reinforce the need for decentralized antivenom distribution and improved healthcare infrastructure in rural areas.

Appropriate and timely treatment is associated with the prevention of complications, improving clinical outcomes. Specific antivenom is the main form of treatment for crotalic envenomation, and it should be administered as quickly as possible [53]. It is important to publicize access to teleconsultation at toxicology information and assistance centers in Brazil, with expert support in providing guidance to local health professionals in managing cases.

Additionally, community actions are of utmost importance to reduce treatment delay. A recent focus group study showed that patients often fail to recognize snakebites as an urgent health concern, with some still believing in ineffective practices, such as attempting to suck venom out of the bite or applying a tourniquet to the affected limb, which may delay treatment and lead to complications [54]. Transportation to the hospital was also seen as an important barrier to timely treatment [54].

It is also important to constantly train the health team in the early identification and treatment and impacts of delayed diagnosis, late propaedeutics and therapy, both for the individual and for the health system. Continuing education, incorporation into the curricular in undergraduate and postgraduate courses of topics related to recording in medical records, mandatory notifications, identification, and diagnosis and conduct towards patients who are victims of snakebite envenomation are also necessary [55].

Occupational exposure remains a critical factor for crotalic envenomation. While most cases were not formally classified as work‐related, incomplete records and informal rural work likely contributed to underreporting. This underscores the importance of accurate occupational history in medical records and educational initiatives promoting the use of personal protective equipment, such as boots and leggings, especially for agricultural workers [54]. It is important to highlight the importance of the health team seeking to properly record occupational incidents, routinely asking about the patient′s work activity, how the event occurred, and whether the patient was working during the activity, in transit to or from work, for example. Due to the retrospective nature of the dataset, detailed information regarding the specific circumstances of the snakebite events (e.g., occupational, recreational, or peridomestic exposure) was not consistently available in the medical records. These factors warrant further investigation in prospective studies.

Systemic complications such as acute renal failure (7.7%), respiratory failure (3.9%), and shock (2.2%) underline the need for immediate treatment. These findings emphasize the importance of health service accessibility and improving awareness through health education on the risk of crotalic envenomation to reduce delays [55]. Furthermore, lack of snake identification increased the odds of severe cases by nearly five times (OR 4.18; 95% CI 2.06–8.77). The lack of identification or proof of the species involved probably hindered and delayed appropriate treatment. Therefore, these findings underscore the need for community education on identifying snakes and their role in guiding treatment.

This study has inherent limitations due to its retrospective cohort design and reliance on data from a single reference center. The information described in the medical records depended on the professionals who conducted the patient′s care; a fact that provided less control over the variables analyzed. Selection and information biases may limit generalizability, as the study population might represent more severe cases. Envenomation is a dynamic process. Clinical manifestations and laboratory abnormalities, specifically neurotoxicity, systemic myolysis, and acute kidney injury, may evolve or deteriorate within the first 48 h of hospitalization. Although classifying patients based on initial presentation may not capture the peak clinical severity reached during the observational period, this approach reflects real‐world emergency triage and the immediate necessity of antivenom administration. By utilizing admission data and opportune reclassification, the study aligns with the critical window for therapeutic decision‐making, where dosage and supportive care are determined by the presenting symptoms to mitigate systemic damage [7]. Additionally, while the high proportion of snakebites in rural areas is well documented, we agree that the increasing occurrence of snakebite incidents in urban areas is an important phenomenon to be studied [56]. In the present analysis, it was neither possible to assure any association, nor tendency changes precisely (as shown in Figure S3). The increase in snakebites in urban areas could be related to multiple factors, such as the effects of climate change and the impacts of human activities (for example, economic and urban development) [57, 58]. However, this is a limitation of the current study, and further research with a longer observation period and different study design would be necessary to explore this hypothesis more thoroughly. Mortality was significantly associated with delays in seeking medical care prior to hospital admission. However, because of the retrospective design, it was not possible to fully distinguish the contribution of prehospital delays from complications that may have occurred during hospitalization.

While the historical series presented in Figure 1 demonstrates a decline in notifications during COVID‐19 pandemic years (specifically, in 2020 and 2021), our study was not designed to assess the impact of COVID‐19 on the epidemiology of crotalic envenomation. The healthcare system, including reference hospitals for antivenom therapy, remained operational during this period. Additionally, Brazil did not implement strict lockdown policies as seen in other countries [59], which may have influenced the dynamics of healthcare access differently. This hypothesis was not tested in our study, and the potential impact of COVID‐19 on the epidemiology of snakebites represents an interesting topic for future research.

This study has also several strengths. By providing a detailed overview of crotalic envenomation cases over a decade, from one of the largest reference centers in toxicology, located in one of the largest emergency hospitals in Latin America [60], this work establishes a robust foundation for understanding the clinical, epidemiological, and laboratory profiles of affected patients, as well as temporal patterns. Unlike previous studies that analyzed shorter periods or nationally aggregated data, our research focuses on Minas Gerais, a state with 853 municipalities, an area comparable with that of Spain or France and home to over 20 million people, encompassing wide socioeconomic and geographic diversity [61–64]. Given its high incidence of snakebites and its wide geographic and socioeconomic diversity [65], Minas Gerais represents a relevant setting for studying crotalic envenomation patterns. Additionally, by identifying key clinical and demographic determinants of severe crotalic envenomation, this study offers actionable insights that can support early risk stratification and inform clinical decision‐making. While our findings are based on a specific region, patterns observed in this high‐incidence area could contribute to a better understanding of snakebite severity in different contexts.

Future research should prioritize interventions aimed at reducing response times, enhancing snake identification, and optimizing clinical management to improve outcomes for affected patients.

## 5. Conclusion

In conclusion, this study highlights the vulnerability of economically active individuals and rural populations to crotalic snakebite envenomation. Seasonality was evident, with a predominance in the summer months, and neuroparalytic manifestations were frequent. Although the median hospital stay was short, systemic complications occurred in 8.3% of cases, and deaths, although rare, accounted for 1.0%. Factors such as age under 20 years and over 50 years, lack of snake identification or confirmation, and delays in treatment exceeding 6 h were significantly associated with severe cases. These findings underscore the need for targeted policies and programs to improve healthcare access in rural areas, alongside awareness campaigns emphasizing the urgency of immediate care following a snakebite.

**Nomenclature**
CAAE: Certificado de Apresentação de Apreciação Ética (Certificate of Presentation of Ethical Review)
CEP‐FHEMIG: Comitê de Ética em Pesquisa da Fundação Hospitalar do Estado de Minas Gerais (Research Ethics Committee of the Hospital Foundation of the State of Minas Gerais)
CI: confidence intervals
CIATox‐MG: Centro de Informação e Assistência Toxicológica de Minas Gerais (Toxicological Information and Assistance Center of Minas Gerais)
CK: creatine phosphokinase
DATATOX: Sistema Brasileiro de Dados de Intoxicações dos Centros de Informação e Assistência Toxicológica (Brazilian Poisoning Data System of the Toxicological Information and Assistance Centers)
ICD‐10: International Classification of Diseases 10th version
INR: international normalized ratio
ISO: International Organization for Standardization
OR: odds ratios
SIGH: Sistema de Informação e Gestão Hospitalar (Hospital Information and Management System)
SINAN/Sinan Net: Sistema de Informações de Agravos de Notificação (*Notifiable Diseases Information System*)

## Author Contributions

Conception: L.R.S. and M.S.M.; study design: L.R.S., M.S.M., and A.A.F.; data collection: V.S.C., V.F.B.N., and P.M.S.R.; analysis and interpretation of the data: A.J.F.R., J.A.C.S., M.S.M., and L.R.S.; drafting of the paper: V.S.C., P.M.S.R., V.F.B.N., L.R.S., M.S.M., and A.J.F.R.; revising the paper critically for intellectual contents and final approval of the version to be published: M.S.M., A.J.F.R., and A.A.F.

## Funding

This study was supported by Fundação de Amparo à Pesquisa do Estado de Minas Gerais (10.13039/501100004901; 1434315/2022‐3) Project: 28967; and Conselho Nacional de Desenvolvimento Científico e Tecnológico (10.13039/501100003593; 310561/2021–3, 465518/2014‐1); National Council for Scientific and Technological Development (CNPq), by the Institute for Health Technology Assessment (IATS/CNPq) (465518/2014‐1).

## Disclosure

This submitted version has been read and approved by all authors.

## Ethics Statement

This study was approved by the Research Ethics Committee of the Minas Gerais State Hospital Foundation–CEP‐FHEMIG, to which Hospital João XXIII belongs (CAAE 03815912.4.0000.5119 and consent to participate declaration dismissal—Committee Opinion, Number 385.243). Given that data were obtained exclusively from medical records and patient de‐identification was ensured, the requirement for informed consent was waived. The authors assure that all the process is aligned to the principles of the Declaration of Helsinki–Ethical Principles for Medical Research Involving Human Participants.

## Consent

The authors have nothing to report.

## Conflicts of Interest

The authors declare no conflicts of interest.

## Supporting information


**Supporting Information** Additional supporting information can be found online in the Supporting Information section. The following supporting information is available for this manuscript and can be accessed in the supplementary material file. These materials provide additional methodological details, reporting checklists, and expanded data visualizations that support the findings presented in the main text: **Supplementary File S1:** STROBE Statement—Checklist of items included in reports of cohort studies. **Figure S1:** Flowchart of patients admitted for suspected crotalic envenomation in Hospital João XXIII (January 2011–December 2022). **Figure S2:** Distribution of in‐hospital length of stay (days) according to number of patients. **Figure S3:** Distribution of cases between rural and urban areas stratified by envenomation year. **Table S1:** Definition of local and systemic complications of ophidian accidents according to the Brazilian notification system (adapted from Sinan Net and the *Manual de Diagnóstico e Tratamento de Acidentes por Animais Peçonhentos*). **Table S2:** Laboratory profile of crotalic accident patients. **Table S3:** Results of the multiple logistic regression model for case severity: likelihood ratio test results and odds ratio estimates with 95% confidence intervals.

## Data Availability

The datasets used and/or analyzed during the current study are available from the corresponding author on reasonable request.
