# Supplementary material for: Decoding Severity in Crotalic Snakebite Cases: Findings From a Decade of Cohort Analysis in Brazil
Source: Biomed Res Int. 2026 Jun 25;2026:7761982. doi: 10.1155/bmri/7761982 (PMC13304233; doi:10.1155/bmri/7761982)
Supplement: Supplementary file 1 — Supporting Information Additional supporting information can be found online in the Supporting Information section. The following supporting information is available for this manuscript and can be accessed in the supplementary material file. These materials provide additional methodological details, reporting checklists, and expanded data visualizations that support the findings presented in the main text: Supplementary File S1: STROBE Statement—Checklist of items included in reports of cohort studies. Figure S1: Flowchart of patients admitted for suspected crotalic envenomation in Hospital João XXIII (January 2011–December 2022). Figure S2: Distribution of in‐hospital length of stay (days) according to number of patients. Figure S3: Distribution of cases between rural and urban areas stratified by envenomation year. Table S1: Definition of local and systemic complications of ophidian accidents according to the Brazilian notification system (adapted from Sinan Net and the Manual de Diagnóstico e Tratamento de Acidentes por Animais Peçonhentos). Table S2: Laboratory profile of crotalic accident patients. Table S3: Results of the multiple logistic regression model for case severity: likelihood ratio test results and odds ratio estimates with 95% confidence intervals. [file BMRI-2026-7761982-s001.docx]

|  | ***Supplementary Material***  **Supplementary File S1:**  ***Strengthening the Reporting of Observational Studies in Epidemiology*** |
| --- | --- |
|  |  |

STROBE Statement—Checklist of items that should be included in reports of ***cohort studies***

|  | **Item No** | **Recommendation** | **Article’s pages No** |
| --- | --- | --- | --- |
| **Title and abstract** | 1 | (*a*) Indicate the study’s design with a commonly used term in the title or the abstract | 1 |
|  |  | (*b*) Provide in the abstract an informative and balanced summary of what was done and what was found | 3 |
| **Introduction** | | |  |
| Background/rationale | 2 | Explain the scientific background and rationale for the investigation being reported | 4,5 |
| Objectives | 3 | State specific objectives, including any prespecified hypotheses | 4 |
| **Methods** | | |  |
| Study design | 4 | Present key elements of study design early in the paper | 5,6 |
| Setting | 5 | Describe the setting, locations, and relevant dates, including periods of recruitment, exposure, follow-up, and data collection | 5 |
| Participants | 6 | (*a*) Give the eligibility criteria, and the sources and methods of selection of participants. Describe methods of follow-up | 5,6, Supplementary material - Figure S1 |
|  |  | (*b*) For matched studies, give matching criteria and number of exposed and unexposed | - |
| Variables | 7 | Clearly define all outcomes, exposures, predictors, potential confounders, and effect modifiers. Give diagnostic criteria, if applicable | 5,6 |
| Data sources/ measurement | 8* | For each variable of interest, give sources of data and details of methods of assessment (measurement). Describe comparability of assessment methods if there is more than one group | 5,6, Supplementary material - Table S1 |
| Bias | 9 | Describe any efforts to address potential sources of bias | 5,6 |
| Study size | 10 | Explain how the study size was arrived at | 5 |
| Quantitative variables | 11 | Explain how quantitative variables were handled in the analyses. If applicable, describe which groupings were chosen and why | 6,7,8,9 |
| Statistical methods | 12 | (*a*) Describe all statistical methods, including those used to control for confounding | 8,9 |
|  |  | (*b*) Describe any methods used to examine subgroups and interactions | 9 |
|  |  | (*c*) Explain how missing data were addressed | 9 |
|  |  | (*d*) If applicable, explain how loss to follow-up was addressed | - |
|  |  | (*e*) Describe any sensitivity analyses | 9 |
| **Results** | | |  |
| Participants | 13* | (a) Report numbers of individuals at each stage of study—eg numbers potentially eligible, examined for eligibility, confirmed eligible, included in the study, completing follow-up, and analysed | 10 |
|  |  | (b) Give reasons for non-participation at each stage | 10 |
|  |  | (c) Consider use of a flow diagram | Supplementary material - Figure S1 |
| Descriptive data | 14* | (a) Give characteristics of study participants (eg demographic, clinical, social) and information on exposures and potential confounders | 10,11,12,13,14 |
|  |  | (b) Indicate number of participants with missing data for each variable of interest | 13,14,15,16,17,18,19 |
|  |  | (c) Summarise follow-up time (eg, average and total amount) | - |
| Outcome data | 15* | Report numbers of outcome events or summary measures over time | 18,19  Supplementary material -Tables S1,S2,S3 |
| Main results | 16 | (*a*) Give unadjusted estimates and, if applicable, confounder-adjusted estimates and their precision (eg, 95% confidence interval). Make clear which confounders were adjusted for and why they were included | 18,19  Supplementary material-Tables S1, S2,S3 |
|  |  | (*b*) Report category boundaries when continuous variables were categorized | 15,16 |
|  |  | (*c*) If relevant, consider translating estimates of relative risk into absolute risk for a meaningful time period | 19, 20  Supplementary material- Table S3 |
| Other analyses | 17 | Report other analyses done—eg analyses of subgroups and interactions, and sensitivity analyses | Supplementary material- Figures 2, 3; Tables S2,S3 |
| **Discussion** | | |  |
| Key results | 18 | Summarise key results with reference to study objectives | 22,23,24 |
| Limitations | 19 | Discuss limitations of the study, taking into account sources of potential bias or imprecision. Discuss both direction and magnitude of any potential bias | 23,24 |
| Interpretation | 20 | Give a cautious overall interpretation of results considering objectives, limitations, multiplicity of analyses, results from similar studies, and other relevant evidence | 22,23,24,25 |
| Generalisability | 21 | Discuss the generalisability (external validity) of the study results | 25 |
| **Other information** | | |  |
| Funding | 22 | Give the source of funding and the role of the funders for the present study and, if applicable, for the original study on which the present article is based | 27 |

* Give information separately for exposed and unexposed groups.

**Note:** An Explanation and Elaboration article discusses each checklist item and gives methodological background and published examples of transparent reporting. The STROBE checklist is best used in conjunction with this article (freely available on the Web sites of PLoS Medicine at http://www.plosmedicine.org/, Annals of Internal Medicine at http://www.annals.org/, and Epidemiology at http://www.epidem.com/). Information on the STROBE Initiative is available at http://www.strobe-statement.org.

**Figure S1.** Flowchart of patients admitted for suspected crotalic envenomation in Hospital João XXIII, between January 2011 to December 2022.

**
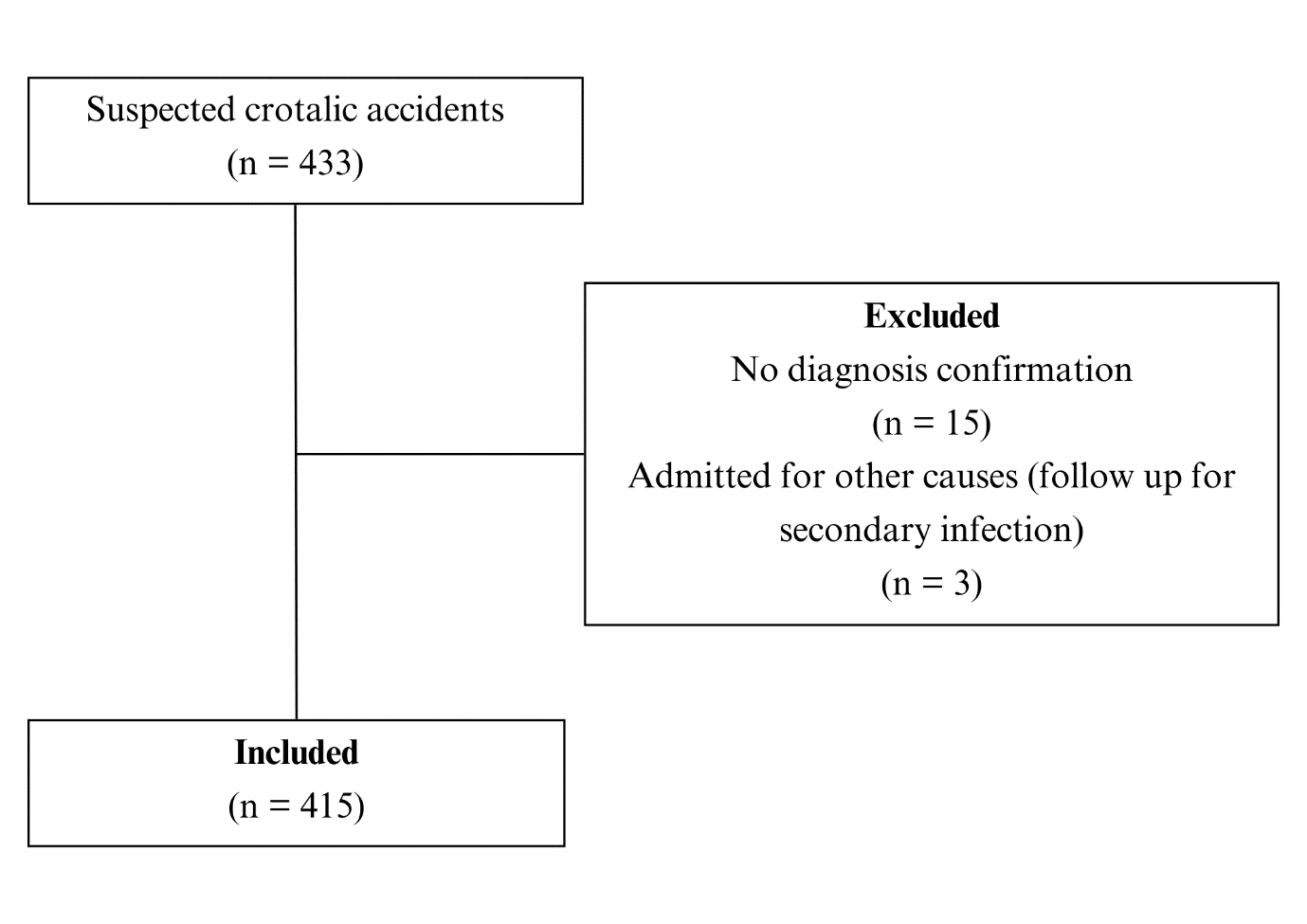
**

**Figure S2.** Distribution of in-hospital length of stay in days according to number of patients.

**
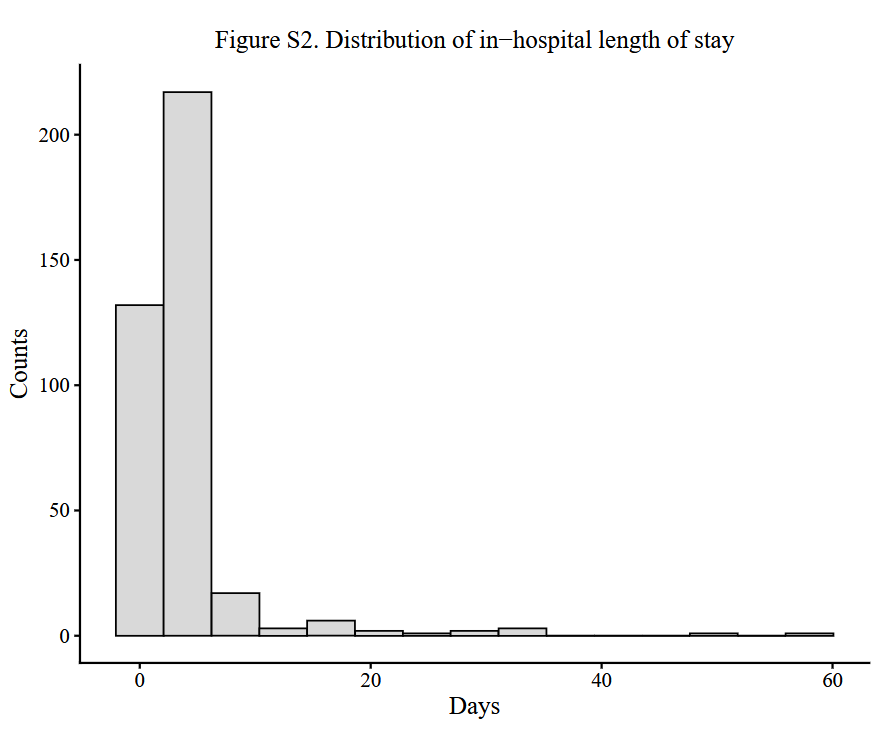
**

**Figure S3.** Distribution of cases between rural and urban areas stratified by envenomation year

**
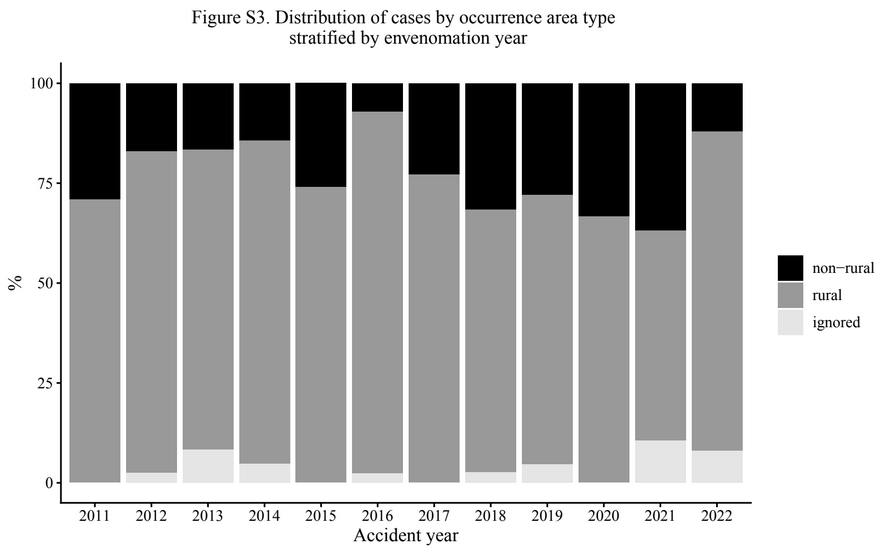
**

**Table S1. Definition of local and systemic complications of ophidian accidents according to Brazilian notification system**

| **Complications** | **Clinical findings** |
| --- | --- |
| **Local** | Persistent local paresthesias, secondary infection, extensive necrosis, compartment syndrome, functional deficit, amputations. |
| **Systemic** | Acute renal failure (due to interstitial nephritis, tubular necrosis and more commonly acute tubular necrosis, secondary to myoglobinuria, shock and hypotension), septicemia, acute respiratory failure/pulmonary edema, shock. |

Source: Table adapted from “*Ficha de investigação acidentes por animais peçonhentos - Sinan Net and Manual de Diagnóstico e Tratamento de Acidentes por Animais Peçonhentos.*” [23].

**Table S2. Laboratory profile of crotalic accident patients**

| **Period** | **Variable** | **n** | **Minimum** | **Q1** | **Median** | **Q3** | **Maximum** |
| --- | --- | --- | --- | --- | --- | --- | --- |
| **Hospital presentation** | Creatinine (mg/dL) | 370 | 0.2 | 0.66 | 0.81 | 0.94 | 5.9 |
|  | CK(U/L) | 379 | 21 | 173 | 397 | 3475 | > 158400 |
|  | INR | 382 | 1 | 1.11 | 1.29 | >10 | > 10 |
|  | Prothrombin activity (%) | 381 | < 10 | < 10 | 69 | 85 | 100 |
|  | Hemoglobin (mg/dL) | 369 | 10 | 12.9 | 14 | 15 | 18.5 |
|  | Fibrinogen (mg/dL) | 377 | <61 | <61 | 68 | 197 | 705 |
|  | Platelets (n/mm³) | 369 | 19000 | 178000 | 215000 | 251000 | 442000 |
| **During hospital stay** | Maximum creatinine (mg/dL) | 368 | 0.2 | 0.72 | 0.86 | 1.01 | 9.24 |
|  | Maximum CK (U/L) | 382 | 27 | 341 | 1128.3 | 14298.5 | > 160000 |
|  | Maximum INR | 382 | 1 | 1.17 | 1.42 | > 10 | > 10 |
|  | Minimum prothrombin activity (%) | 382 | < 10 | < 10 | 61 | 78 | 100 |
|  | Minimum hemoglobin (mg/dL) | 369 | 4.4 | 12.2 | 13.3 | 14.3 | 17.1 |
|  | Minimum fibrinogen (mg/dL) | 378 | <61 | <61 | <61 | 159,5 | 705 |
|  | Minimum platelets (cels/mm³) | 369 | 19000 | 160000 | 192000 | 233000 | 442000 |

CK: Creatine phosphokinase; INR: international normalized ratio; Q1: first quartile; Q3: third quartile

**Table S3.** Results of the multiple logistic regression model for case severity: results of the likelihood ratio test and estimates of the odds ratios of severe cases with their respective 95% confidence intervals

| **Variable** | **Categories** | **Multiple logistic regression** | |
| --- | --- | --- | --- |
|  |  | **P-value*** | **Odds ratio (95% CI)** |
| Sex | Female | 0.960 | 1 |
|  | Male |  | 1.01 (0.58; 1.79) |
| Age (years) | [20,50) | <0.001 | 1 |
|  | [ 0,20) |  | **2.99 (1.65; 5.50)** |
|  | [50,90] |  | **1.79 (1.08; 2.96)** |
| Time until hospital admission (hours) | < 6 | <0.001 | 1 |
|  | ≥ 6 |  | **4.04 (2.46; 6.72)** |
|  | Not informed |  | 0.46 (0.10; 1.64) |
| Identification of the snake | Identified | <0.001 | 1 |
|  | Identified, but not confirmed |  | **2.60 (1.54; 4.44)** |
|  | Non identified |  | **4.18 (2.06; 8.77)** |
|  | Not informed |  | 1.51 (0.76; 2.98) |
| Zone of occurrence | Urban | 0.062 | 1 |
|  | Rural |  | 1.48 (0.83; 2.70) |
|  | *Periurban* |  | 0.36 (0.07; 1.33) |
|  | Not informed |  | 0.80 (0.20; 2.84) |
| * Likelihood ratio test for marginal effect of variable  CI: Confidence interval | | | |
